# Role of the Arylhydrocarbon Receptor (AhR) in the Pathology of Asthma and COPD

**DOI:** 10.1155/2012/372384

**Published:** 2012-01-29

**Authors:** Takahito Chiba, Junichi Chihara, Masutaka Furue

**Affiliations:** ^1^Department of Dermatology, Graduate School of Medical Sciences, Kyushu University School of Medicine, 3-1-1, Maidashi, Higashi-Ku, Fukuoka 812-8582, Japan; ^2^Department of Clinical and Laboratory Medicine, Akita University School of Medicine, Akila 010-8502, Japan; ^3^Research and Clinical Center for Yusho and Dioxin, Kyushu University Hospital, Fukuoka 812-8582, Japan

## Abstract

The dioxins and dioxin-like compounds in cigarette smoke and environmental pollutants modulate immunological responses. These environmental toxicants are known to cause lung cancer but have also recently been implicated in allergic and inflammatory diseases such as bronchitis, asthma, and chronic obstructive pulmonary disease (COPD). In a novel pathway of this response, the activation of a nuclear receptor, arylhydrocarbon receptor (AhR), mediates the effects of these toxins through the arachidonic acid cascade, cell differentiation, cell-cell adhesion interactions, cytokine expression, and mucin production that are implicated in the pathogenesis and exacerbation of asthma/COPD. We have previously reported that human bronchial epithelial cells express AhR, and AhR activation induces mucin production through reactive oxygen species. This review discusses the role of AhR in asthma and COPD, focusing in particular on inflammatory and resident cells in the lung. We describe the important impact that AhR activation may have on the inflammation phase in the pathology of asthma and COPD. In addition, crosstalk of AhR signaling with other ligand-activated transcription factors such as peroxisome proliferator-activated receptors (PPARs) has been well documented.

## 1. Introduction

Both allergic asthma and COPD are defined as airway inflammatory diseases; however, the inflammatory mechanism is different for each disease. Nocuous agents such as PCBs, B[a]P, and dioxin-like compounds in cigarette smoke and environmental pollutants have the potential to induce inflammation or exacerbate chronic bronchitis, asthma, COPD, and lung cancer [1–4]. In addition to airway epithelial cells, many inflammatory cells, including Th2 cells, eosinophils, and basophils, play a major pathophysiological role in asthma and COPD [5–8]. Cigarette smoke and environmental pollutants activate these inflammatory cells, and they contribute to the activation of growth factors and cytokines. For example, exposure to some types of noxious agents increases the rate of TGF-*α*, TGF-*β*, IL-1*β*, IL-6, IL-8, and IFN-*γ* gene expression [9–12]. While the molecular signaling mechanism for this transcriptional modulation of cytokines remains to be determined, it has been recently recognized that these effects are mainly mediated through the binding of noxious agents to the AhR. All major human cell types express AhR, including pulmonary tissue [13, 14]. The liver, adipose tissue, and skin are the major storage sites of AhR ligands in humans [15]. These AhR ligands are also concentrated in bronchial epithelial cells, suggesting that the respiratory system is sensitive to AhR ligands [16]. 

The AhR is a ligand-activated transcription factor, and after ligation of dioxins to the AhR, the receptor translocates from the cytosol to the nucleus, where it heterodimerizes with the ARNT. It then binds to a DRE, an enhancer sequence of several drug-metabolizing enzymes, such as CYP1A1 [17]. AhR-induced CYP1A1 activation is important for detoxication. CYPs convert B[a]P and dioxin-like compounds into physiologic metabolites that exert effects on cell growth, differentiation, and migration. A number of researchers have demonstrated the molecular aspects of the AhR pathway by using selective agonists such as TCDD or B[a]P among PAHs.

In this review article, we summarize current findings regarding the functional role of AhR molecules in airway inflammation and focus on bronchial epithelial cells, fibroblasts, granulocytes, and lymphocytes. Understanding the effects of AhR on these cells would be a breakthrough in our understanding of the pathology and treatment of asthma and COPD. 

## 2. Airway Inflammatory Effect through AhR Activation in Asthma and COPD

### 2.1. Airway Epithelial Cells

**Figure 1:**
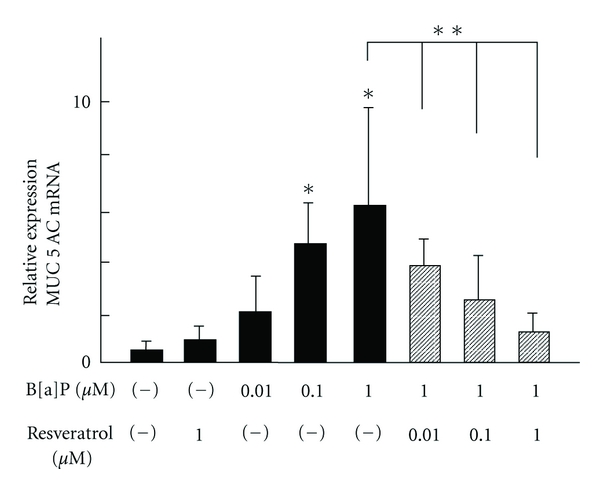
Effects of AhR agonist B[a]P on MUC5AC mRNA level in NCI-H_292_ after 12 h of incubation. MUC5AC was measured by real-time RT-PCR. B[a]P induced MUC5AC mRNA expression in dose-dependent manner. Pretreatment with AhR antagonist, resveratrol, inhibited AhR-induced MUC5AC upregulation. Data are expressed as means ± SD (*n* = 6). *P < 0.05 versus control (medium alone). **P < 0.05 versus B[a]P 1 *μ*M.

Airway epithelial cells are able to modify allergic airway inflammation by virtue of their ability to produce a variety of inflammatory mediators [18, 19]. One such mediator is the moderate bronchial mucin-containing mucus, which normally protects the airway from exogenous substances. Hypermucosis in the airway, however, is associated with several respiratory diseases, including asthma and COPD. Mucus hypersecretion in the airway increases coughing and expectoration of sputum. Clara cells in the airway can secrete a wide variety of glycoproteins, such as mucins and SP-D, and are very sensitive to AhR stimulation [20, 21]. Wong et al. recently have reported TCDD, an AhR agonist, increased expression of inflammatory cytokines, MUC5AC, and MMPs via AhR signaling in a Clara-cell-derived cell line [21]. Mucus production is typically mediated by cytokine or lipid mediator release, or an increase of ROS [22–24]. Studies using AhR agonists and inhibitors have demonstrated that AhR activation induces the production of cytokines such as TGF-*α*, TNF-*α*, and MMP through receptors in human hematocytes and epithelial cells [21, 25–27]. Wong et al. also reported an increase of COX-2 and IL-1*β* mRNA expression in response to AhR activation [21]. The production of prostanoids such as PGE_2_, which is derived from COX-2, can activate mucin production in the airway [22]. Although prostaglandins derived from COX-2 pathway activation may be responsible for AhR-induced mucin production in the bronchial epithelial cells, the mechanism of their action remains to be determined. Therefore, it is of paramount interest to investigate the mechanism by which AhR activation induces mucin production. In an earlier study, we reported findings similar to those by Wong et al. In our study, we found that AhR activation upregulates the expression of MUC5AC and mucin secretion in a NCI-H292 cell line that was derived from a bronchiolar Clara cell [14] (Figure 1). Moreover, we concurrently showed that AhR activation induced ROS generation, and the antioxidant agent NAC inhibited B[a]P-induced MUC5AC upregulation. Kopf and Walker also demonstrated that TCDD-induced AhR activation increased ROS levels in endothelial cells [29]. Another prostaglandin, PGD_2,_ is synthesized from arachidonic acid via the catalytic activities of COX in epithelial cells and mast cells. It is released into the airway following an antigen challenge during an acute allergic response [30]. PGD_2_ induces chemotaxis of Th2 cells, eosinophils, and basophils as a consequence of the activation of its receptors [31]. This suggests that PGD_2_ promotes inflammation in allergic asthma. Prostaglandins that are derived from COX-2 pathway activation and ROS that are induced by AhR activation are the major inflammatory mediators capable of inducing mucin production, inflammatory cell chemotaxis, or inflammatory cell activation. Therefore, increased levels of prostaglandins and ROS, either directly or through the formation of lipid peroxidation products, may enhance the inflammatory response in both asthma and COPD.

**Figure 2:**
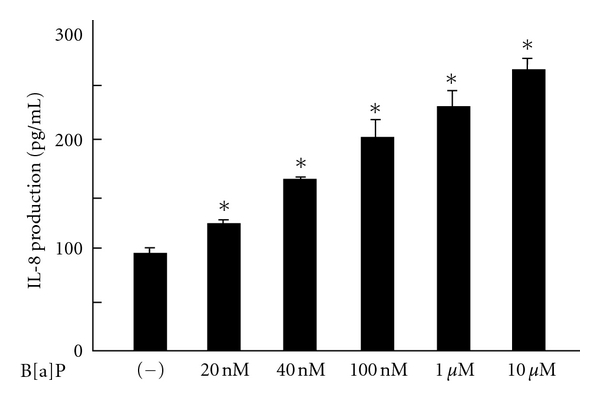
Normal human epidermal keratinocytes (NHEKs) were exposed to B[a]P at various concentrations for 24 h, and IL-8 production in the culture supernatant was measured. B[a]P induced IL-8 production in a dose-dependent manner. Data are expressed as means ± SD (*n* = 3). *P < 0.05 versus control (medium alone).

Neutrophils isolated from peripheral blood and BAL fluid of asthmatic patients generate more ROS than cells from normal patients. Additionally, the production of ROS correlates with the degree of airway hyperresponsiveness [32, 33]. Neutrophils and macrophages are also known to migrate into the lungs of COPD patients [34, 35]. Indeed, the neutrophils that mediate ROS-induced injury to the airway epithelium are responsible for hyperresponsiveness in human peripheral airways, suggesting that neutrophils play an important role in the pathogenesis of asthma and COPD [36]. AhR-derived inflammatory mediators in airway epithelial cells, such as IL-8 and leukotriene B4, may have a chemotactic effect. We previously confirmed that normal human epidermal keratinocytes (NHEKs) enhanced IL-8 production through AhR activation [37] (Figure 2). Martinez et al. demonstrated that IL-8 gene expression was upregulated by TCDD in A549 cells from a bronchial epithelial cell line [38]. However, they could not detect IL-8 production at the protein level in airway epithelial cells. We were also unable to detect IL-8 production from AhR-activation in NCI-H292 cells using ELISA analysis (data not shown). Although it is not clear that AhR directly modulates NF-*κ*B, the induction of a transcription factor for IL-8, tumor necrosis factor, or IL-1*β* by AhR activation might impact IL-8 production in airway epithelial cells [21, 25, 26, 39]. 

**Figure 3:**
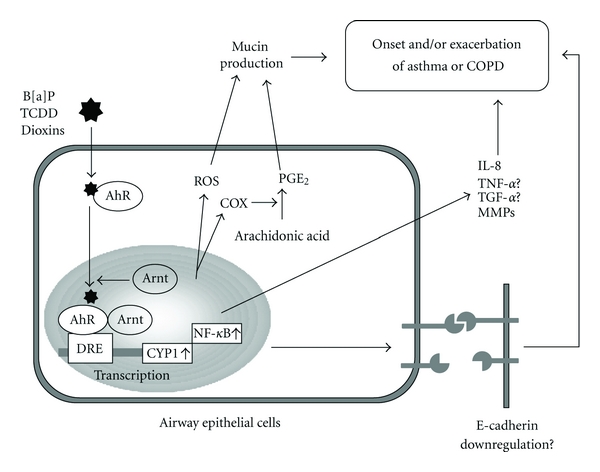
Schematic diagram of the proposed crosstalk AhR-signaling pathway and inflammatory effects in airway epithelial cells.

Cell-cell contact molecules in the airways create a barrier that plays an important role in the defense against bacteria. Loss of expression of cell-cell contact molecules, such as E-cadherin, reduces the ability of epithelial cells to function as a barrier and may increase the allergic response and susceptibility to infection. Indeed, E-cadherin and *α*-catenin interacted with cytosolic domain of the cadherin expression are significantly lower in asthmatic than in nonasthmatic subjects [40]. AhR also regulates the expression of adhesion molecules and consequently controls cell-cell contact. Exposure to TCDD from a human breast cancer cell line downregulates E-cadherin expression [41]. Using rat liver epithelial cells, Dietrich et al. demonstrated that TCDD exposure inhibits the expression of *γ*-catenin, which links E-cadherin to actin filaments [42]. We hypothesize that several pathways may be involved in the production of inflammatory cytokines and mucus in asthma and COPD, as illustrated in Figure 3.

### 2.2. Fibroblast or Airway Smooth Muscle

Chronic asthmatic patients who are unresponsive to treatment experience progressive and irreversible changes in pulmonary function. These changes, known as “airway remodeling,” are associated with structural alterations, such as subepithelial fibrosis, smooth muscle or goblet cell hyperplasia, and airway hyperresponsiveness [43]. In chronic asthma patients, fibrosis is due to increased deposition of extracellular matrix. Increases in airway smooth muscle mass are thought to be caused by faster proliferation, mitogenic, or inflammatory stimuli [44]. Some of the factors contributing to these effects are TGF, FGF, EGF, and PDGF. TGF-*β* is one of these contributors and is a major effector cytokine that can increase deposition by fibroblasts and airway smooth muscle hypertrophy. Guo et al. reported that levels of RNA for TGF-*β*2 and TGF-*β*2-related genes increased in AhR-knockout smooth muscle cells [45]. This suggests that AhR may repress the TGF-*β*- signaling pathway, resulting in an anti-inflammatory effect unlike in rodent lung cells. On the other hand, cigarette smoke, via AhR, can induce cyclooxygenase and PGE_2_ in human lung fibroblasts [46]. PGE_2_ significantly enhances cigarette smoke extract-treated neutrophil chemotaxis and adhesion to airway epithelial cells [47]. In fact, the concentration of PGE_2_ in the sputum of COPD patients is correlated with the number of infiltrating neutrophils [47]. Neutrophil activation through AhR signaling plays a causal role in pathogenesis and exacerbation of COPD.

### 2.3. Granulocytes with Focus on Eosinophils

**Figure 4:**
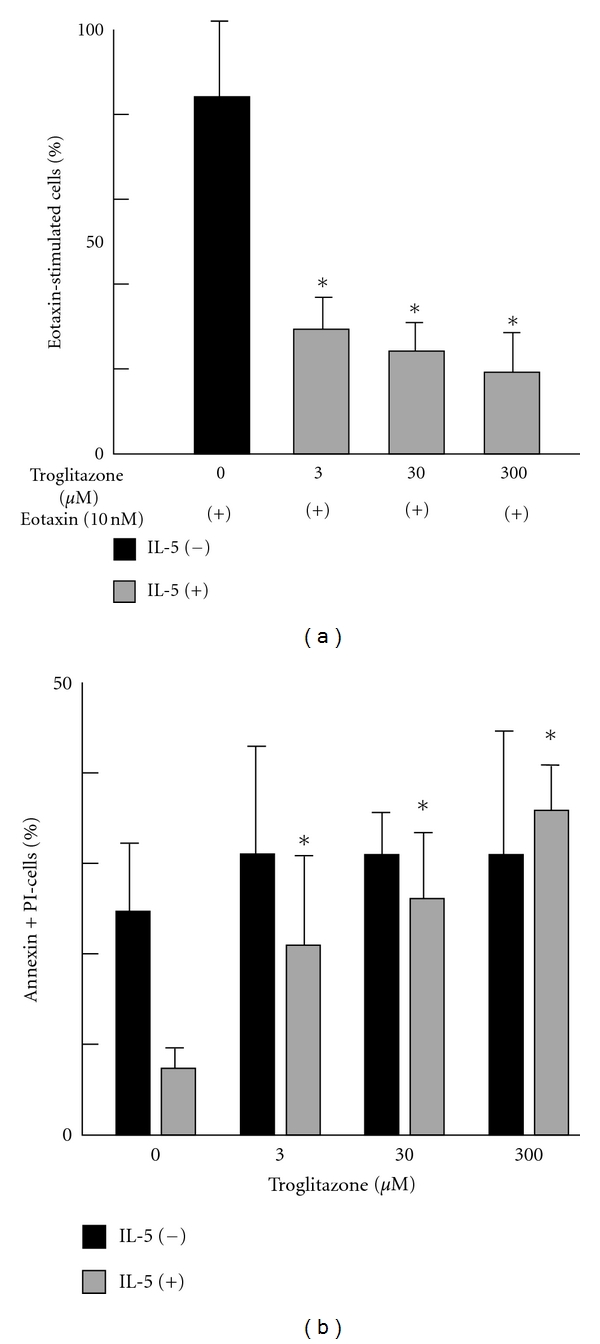
(a) Effect of PPAR*γ* agonist troglitazone on eosinophil chemotaxis stimulated with eotaxin. Purified eosinophils were preincubated with increasing concentrations of troglitazone for 1 h. Migration assays were performed using Boyden chambers. Chemotactic response to eotaxin alone was considered to be 100%, and reactions to lower concentrations are presented relative to eotaxin alone. Data are expressed as mean ± SD. Troglitazone inhibited the eotaxin-directed eosinophil chemotaxis in a dose-dependent manner (*n* = 4). *P < 0.05 versus eotaxin alone. (b) Effect of troglitazone on eosinophil survival determined by staining with Annexin V-FITC and propidium iodine. Eosinophils were incubated with and without troglitazone in the presence of 1 ng/mL IL-5 for 48 h. Eosinophils were treated with Annexin V to stain early-phase apoptotic cells and with propidium iodine (PI) to stain the late-phase cells. The bar graph shows a dose-dependent effect of troglitazone on IL-5-induced eosinophil survival (*n* = 4). Data are expressed as mean ± SD. *P < 0.05 versus without troglitazone.

Eosinophils play an essential role in the pathology of asthma because they contribute to tissue injury, vascular leakage, mucus secretion, and tissue remodeling by releasing cytotoxic granule proteins, ROS, and lipid mediators [48]. Because eosinophils are the final effector cells in allergic inflammation, it is important to study the process by which nuclear receptors, such as AhR, activate eosinophils in order to understand the pathogenesis of allergic diseases. For example, PPARs are among the important ligand-activated transcription factors that regulate the expression of genes involved in many cellular functions, including differentiation, immune responses, and inflammation [49, 50]. The PPAR subfamily consists of 3 isotypes: PPAR*α*, PPAR*β*/*δ*, and PPAR*γ*, all of which have been identified in eosinophils. These nuclear receptors form heterodimers with retinoid X receptors, bind to a specific DNA sequence (PPRE), and activate target gene transcription. *In vivo* and *in vitro* evidence suggests that PPAR*α* and PPAR*γ* expression in granulocytes and dendritic cells plays a critical role as an inflammatory suppressive regulator in allergic diseases. Treatment with the PPAR*γ* agonist, rosiglitazone, decreases the clinical severity of skin lesions in atopic dermatitis and airway inflammation in asthmatic patients [51, 52]. We previously demonstrated that the PPAR*γ* agonist troglitazone reduced IL-5-stimulated eosinophil survival, eotaxin-directed eosinophil chemotaxis, and functional augmentation of eosinophil adhesion in a concentration-dependent manner. These changes occurred without reducing the quantitative expression of *β*2 integrins [53, 54] (Figure 4). It has been lately shown that PPAR*γ* induction is suppressed during the activation of the AhR by TCDD [55]. In addition, Cho et al. demonstrated that CYP1B1 upregulation induced the inhibition of AhR expression in 10T1/2 cells derived from preadipocyte lines. Moreover, the reduced AhR expression was accompanied by an increase in PPAR*γ* expression [56]. These results suggest that the AhR signal may repress migration, degranulation, and cellular adhesion of eosinophils. This may impair the antiallergic effects induced by PPAR*γ*. We were able to confirm AhR expression in human eosinophils using RT-PCR (data not shown). Clarification of the interaction between AhR and PPAR*γ* signals should broaden our understanding not only of the functional role of eosinophils but also of asthma regulation.

### 2.4. Lymphocytes

Allergic asthma is associated with disruption of the immune system, particularly an imbalance of Th1 and Th2 cells. It is well known that Th2 cells play a key role in the regulation of inflammatory reactions through the release of Th2 cytokines. AhR is known to exert an influence on allergic immunoregulation. In fact, Tauchi et al. reported that mice with constitutive AhR activation developed severe skin lesions that were similar to the lesions seen in atopic dermatitis. The lesions were accompanied by high serum levels of IgE and increased production of IL-4 and IL-5 from stimulated splenic lymphocytes [57]. In addition, AhR expression in splenic B cells was enhanced by the presence of lipopolysaccharide, which is known to exacerbate asthma and COPD [58]. PAH and TCDD increase IgE production in cocultures with purified B cells [59]. These results provide further evidence that AhR may play a complex role in the humoral immunological balance in airway allergic pathogenesis. 

Th17 cells have been recently classified as a subtype of helper T cells that are characterized by the production of IL-17 [60]. AhR activation promotes the development of Th17 cells and results in increased pathology in animal models of multiple sclerosis [61]. Th17 cells found in the skin, gastrointestinal tract, and bronchial tubes are involved in inflammatory conditions, such as inflammatory bowel disease and asthma [62]. The IL-17 produced by Th17 cells is a potent activator of NF-*κ*B, thereby, increasing the levels of inflammatory cytokines such as IL-8, IL-6, TNF-*α*, G-CSF, and GM-CSF [63]. Therefore, although Th17 plays a role in regulating neutrophil and macrophage inflammation, it is not known whether IL-17 induced by AhR activation contributes to the development of asthma or COPD. Clinically, IL-17 levels in BAL fluid, sputum, and peripheral blood from patients with allergic asthma are higher than those in healthy controls [64, 65]. A knockout mouse model of the IL-17 receptor showed reduced OVA-induced airway hyperresponsiveness and eosinophil infiltration. Additionally, the levels of IgE and Th2 cytokines in knockout mice were not as highly elevated as they were in wild-type mice [66]. Furthermore, stimulation with IL-17 increased the concentration of biologically active MMP-9 in mouse airways. IL-17 protein, as represented by neutrophilic inflammation, has been detected in COPD patients, but at a lower level than observed in asthma patients [67]. Human lymphocytes, however, may behave differently. For example, AhR agonists appear to favor IL-22 but not IL-17 production in humans [68]. These studies suggest a role of AhR-induced Th17 in promoting allergic or inflammatory airway diseases, but there are interesting differences between human and mouse T cells. These differences suggest that the response to AhR activation may vary according to cell type, maturation, and differentiation process.

## 3. Conclusion

We reviewed studies on the relationship between AhR function and airway inflammation, as it is important in the initial phase of asthma/COPD. In addition to studying the toxicological effects, we wish to promote studies focused on the immune regulation of endogenous AhR pathways. Moreover, it seems increasingly apparent that AhR acts by competing with other nuclear receptors in a complex manner. Further investigation may yield a novel treatment strategy for AhR-associated lung diseases.

## Abbreviations

PCBs: Polychlorinated biphenyls
B[a]P: Benzopyrene
COPD: Chronic obstructive pulmonary disease
TGF-*α*: Transforming growth factor alpha
AhR: Arylhydrocarbon receptor
ARNT: AhR nuclear translocator
DRE: Dioxin response element
CYP1A1: Cytochrome P450 1A1
TCDD: 2,3,7,8-tetrachlorodibenzo-*p*-dioxin
ROS: Reactive oxygen species
TNF-*α*: Tumor necrosis factor alpha
MMP: Matrix metalloproteases
COX-2: Cyclooxygenase-2
PGE_2_: Prostaglandin E_2_
PGD_2_: Prostaglandin D_2_
MUC5AC: Oligomeric mucus/gel forming
BAL: Bronchoalveolar lavage
PPARs: Peroxisome proliferator-activated receptors
PPRE: PPARs response element
IgE: Immunoglobulin E
G-CSF: Granulocyte colony-stimulating factor
FGF: Fibroblast growth factor
EGF: Epidermal growth factor
PDGF: Platelet-derived growth factor.
